# 

**Published:** 1905-10-15

**Authors:** 


### ANNOUNCEMENTS.

EVANS CROWN, BRIDGE AND PORCELAIN WORK. A new edition of this work is just out from the publishing department of the S. S. White Dental Manufacturing Co. The work is standard, as well as the original of its kind. The present edition is more than a reprint; as much new matter has been added, including porcelain crown and bridge technics, which brings the work up to date. The old text has been revised in many places and much new matter has been introduced. Some of the old methods of crown making might have been dropped as they are not often used, and much more space might have been given to the later developments in making the seamless crowns, so useful in making bridge work in the posterior teeth. The porcelain jacket crown, the most artistic crown ever invented, should have received adequate attention, as it is destined to supplant the metal jacket crown for all exposed teeth in the better class of practice. As was to be expected, the book is well made and is a valuable addition to our literature.

SOUTHERN CALIFORNIA DENTAL ASSOCIATION.—The Southern California Dental Association will hold its eighth annual session in Los Angeles, Nov. 6th to 8th, 1905. A cordial invitation is extented to the profession.

CHAS. M. BENBROOK, *Sec'y.*
455 S. Broadway, Los Angeles.

SEVENTH AND EIGHTH DISTRICT DENTAL SOCIETIES OF THE STATE OF NEW YORK,—The Seventh and Eighth District Dental Societies of New York will hold a union meeting at the Osborn House, Rochester, October 31st to November 2nd, 1905. Mark these dates off in your appointment book and attend. It will be time well spent. If you have anything of interest communicate with the business committee, C. F. Bunbury, Chairman, 62 State Street, Rochester; J. W. Graves, 32 Triangle Building, Rochester; S. Eschelman, 421 Franklin Street, Buffalo; D. H. Young, Attica. Clinic Committee, G. G. Burns and F. Messerschmitt, Rochester.

J. T. DEVOL, 81 years old, formerly a dentist at Parkersburg, W. Va., died August 13th, 1905.

THE OHIO STATE DENTAL SOCIETY will hold its fortieth annual meeting at Columbus, December 5th, 6th and 7th, 1905. Dr. H. C. Brown, 185 E. State Street, is chairman of of the program committee and Dr. A. O. Ross, 807 N. High Street, is chairman of the clinic committee. Get into correspondence with these gentlemen if you have anything to offer.

THE OHIO STATE BOARD OF DENTAL EXAMINERS will hold its regular semi-annual meeting November 28th, 29th and 30th, 1905, at the Hartman Hotel, in Columbus, O. Applications for examinations should be filed with the secretary not later than November 18th. For full particulars write to Dr. H. C. Brown, 185 E. State Street, Columbus, O.

—✦—

OFFICERS NEW JERSEY STATE SOCIETY. The following officers were elected at the last meeting: President, J. E. Duffield; Secretary, Charles E. Meeker; Chairman Executive and Essay Committees, M. R. Brinkman; Essay Committee, W. Woolsey.

—✦—

THE JENKINS SOCIETY. This is a new organization, formed September 1st, with Dr. W. I. Brigham, of South Framingham, Mass., as President, and Dr. B. C. Guile, of Penn Yan, N. Y., as Secretary and Treasurer. The object is to disseminate Porcelain Literature and the dues of one dollar per year are to be used for this purpose. Any dentist of any country who is an ethical practitioner, can become a member.

—✦—

A DENTAL MANUAL OF THERAPEUTICS AND MATERIA MEDICA. Detroit, Parke, Davis & Co., Publishers. Cloth, 68 pages.

This handy little book contains a vast amount of information of a matter-of-fact sort. The section on Dental Therapy is an alphabetical arrangement of the various diseases most commonly met with in practice. Under the several sections are useful suggestions as to the most acceptable preventive and remedial treatment, each presented in a few terse sentences. The section on Dental Materia Medica is composed of a comprehensive list of the best and latest drugs and preparations, alphabetically arranged for ready reference and supplemented with a "Classification of Drugs According to Their Uses," as analgesics, astringents, bleaching agents, etc. The whole constitutes a valuable and useful compend that any dental practitioner will find use for daily.

